# Stirred, not shaken: genetic structure of the intermediate snail host *Oncomelania hupensis robertsoni *in an historically endemic schistosomiasis area

**DOI:** 10.1186/1756-3305-4-206

**Published:** 2011-10-26

**Authors:** Anne-Kathrin Hauswald, Justin V Remais, Ning Xiao, George M Davis, Ding Lu, Margaret J Bale, Thomas Wilke

**Affiliations:** 1 Department of Animal Ecology & Systematics, Justus Liebig University, Heinrich-Buff-Ring 26-32 IFZ, D-35392 Giessen, Germany; 2 Department of Environmental Health, Rollins School of Public Health, Emory University, 1518 Clifton Rd. NE, Atlanta, GA 30322, USA; 3 Program in Population Biology, Ecology and Evolution, Graduate Division of Biological and Biomedical Sciences, Emory University, 1510 Clifton Rd, Atlanta, GA 30322, USA; 4 Institute of Parasitic Diseases, Sichuan Center for Disease Control and Prevention, Chengdu, Sichuan 610041, People's Republic of China; 5 Department of Microbiology and Tropical Medicine, George Washington University Medical Center, 745 Ross Hall, 2600 Eye St., N.W., Washington, D.C. 20037, USA

**Keywords:** China, Sichuan Province, *Oncomelania hupensis robertsoni*, schistosomiasis japonica, coevolution, AFLP, cytochrome *c *oxidase subunit I (COI), phylogeny, phylogeography, watersheds

## Abstract

**Background:**

*Oncomelania hupensis robertsoni *is the sole intermediate host for *Schistosoma japonicum *in western China. Given the close co-evolutionary relationships between snail host and parasite, there is interest in understanding the distribution of distinct snail phylogroups as well as regional population structures. Therefore, this study focuses on these aspects in a re-emergent schistosomiasis area known to harbour representatives of two phylogroups - the Deyang-Mianyang area in Sichuan Province, China. Based on a combination of mitochondrial and nuclear DNA, the following questions were addressed: 1) the phylogeography of the two *O. h. robertsoni *phylogroups, 2) regional and local population structure in space and time, and 3) patterns of local dispersal under different isolation-by-distance scenarios.

**Results:**

The phylogenetic analyses confirmed the existence of two distinct phylogroups within *O. h. robertsoni*. In the study area, phylogroups appear to be separated by a mountain range. Local specimens belonging to the respective phylogroups form monophyletic clades, indicating a high degree of lineage endemicity. Molecular clock estimations reveal that local lineages are at least 0.69-1.58 million years (My) old and phylogeographical analyses demonstrate that local, watershed and regional effects contribute to population structure. For example, Analyses of Molecular Variances (AMOVAs) show that medium-scale watersheds are well reflected in population structures and Mantel tests indicate isolation-by-distance effects along waterways.

**Conclusions:**

The analyses revealed a deep, complex and hierarchical structure in *O. h. robertsoni*, likely reflecting a long and diverse evolutionary history. The findings have implications for understanding disease transmission. From a co-evolutionary standpoint, the divergence of the two phylogroups raises species level questions in *O. h. robertsoni *and also argues for future studies relative to the distinctness of the respective parasites. The endemicity of snail lineages at the regional level supports the concept of endemic schistosomiasis areas and calls for future geospatial analyses for a better understanding of respective boundaries. Finally, local snail dispersal mainly occurs along waterways and can be best described by using cost distance, thus potentially enabling a more precise modelling of snail, and therefore, parasite dispersal.

## Background

The parasite species of the trematode genus *Schistosoma *cause human schistosomiasis, one of the most prevalent parasitic diseases in the world, infecting more than 200 million people and leading to a substantial burden of disease [1]. *Schistosoma japonicum*, the causal agent of the disease common in East and Southeast Asia, remains a public health threat for millions of people living in the tropical and subtropical zones of China [2]. Though overall prevalence and intensity of infection have decreased greatly in recent decades [3], cases of re-emergence remain of concern [4,5]. The considerable medical importance of *S. japonicum *has spurred numerous parasitological, ecological and genetic studies. Genetic variation between strains of *S. japonicum *from widely separated geographical areas, for example, have been reported based on allozyme, nucleotide sequence, microsatellite and single nucleotide polymorphism analyses [6-15]. Indeed, the genetic distance between some *S. japonicum *populations is so immense as to indicate that they represent distinct taxa [10]. In particular, researchers consider that parasites from Sichuan Province, which are transmitted by the nominal snail subspecies *Oncomelania hupensis robertsoni*, possibly belong to a separate strain [10-12].

Given the demonstrated close co-evolutionary relationships between *S. japonicum *and its intermediate snail host *O. hupensis *ssp. [16], there is an increased interest in understanding snail phylogenetics and population structure. The importance of the snail host for comprehending disease transmission is further reinforced by three principal findings:

1) *Oncomelania hupensis *is the sole intermediate host for *Schistosoma japonicum*: unlike *S. mansoni *and *S. haematobium*, host switching does not appear to occur.

2) Given the close genetic interactions between snail and parasite in terms of co-evolutionary relationships, a snail population likely reflects population genetic parameters of the parasite and vice versa [17,18].

3) Genetically diverse snail populations appear to be more susceptible to infection with *S. japonicum *than homogeneous populations [19].

Molecular and morphological analyses, together with breeding experiments and biogeographic studies of *O. hupensis *indicate that there are three subspecies on the mainland of China [16,20-22]. *Oncomelania h. hupensis *primarily occurs in the Yangtze River drainage below the Three Gorges; it has spread to Guangxi Province via the Grand Canal from Hunan (note that some authors consider the latter populations to belong to a distinct subspecies, *O. h. guangxiensis *[23,24]). *Oncomelania h. tangi *is restricted to Fujian Province along the coast, and *O. h. robertsoni *has a patchy distribution in Sichuan and Yunnan Provinces above the Three Gorges. However, subspecies validity and assignment remains controversial [24,25], including the western subspecies *O. h. robertsoni*. Genetically, it is highly distinct from all other known subspecies of *O. hupensis *[16,22,26,27], raising questions about whether it deserves full species status. Moreover, based on mitochondrial (mt) DNA sequence data, the existence of at least two major phylogroups within *O. h. robertsoni *was demonstrated, with pairwise K2P-distances for two mtDNA genes of up to 0.12 (= 12%) [26]. Because such a high divergence typically reflects genus-level relationships within the snail superfamily Rissooidea [28], it was initially not clear whether the patterns observed are due to the presumed long evolutionary history of this subspecies [16] or are simply artefacts. Note that *O. hupensis *is a dioecious species (i.e., an individual specimen is distinctly male or female). Thus selfing cannot explain the overall high diversity within this species either.

Only recently, an independent study based on DNA sequencing data of a nuclear (nc) gene confirmed the existence of two phylogroups [27]. However, the regional distribution of these groups is not well understood. Moreover, possible co-evolutionary implications of these two genetically and sexually isolated snail host taxa for the genetic structure of *S. japonicum *remain unknown. At the same time, little knowledge exists about smaller-scale population structures in *O. h*. *robertsoni*, such as those within schistosomiasis transmission areas. As a consequence, local effects of barriers (such as watershed boundaries or mountain ranges) and means of dispersal (such as along water networks or bird-mediated) on snail and thus parasite distributions are poorly understood. Given these knowledge gaps, there is a need for better understanding of the phylogroup distribution in *O. h. robertsoni* and regional population structures in time and space.

**Figure 1:**
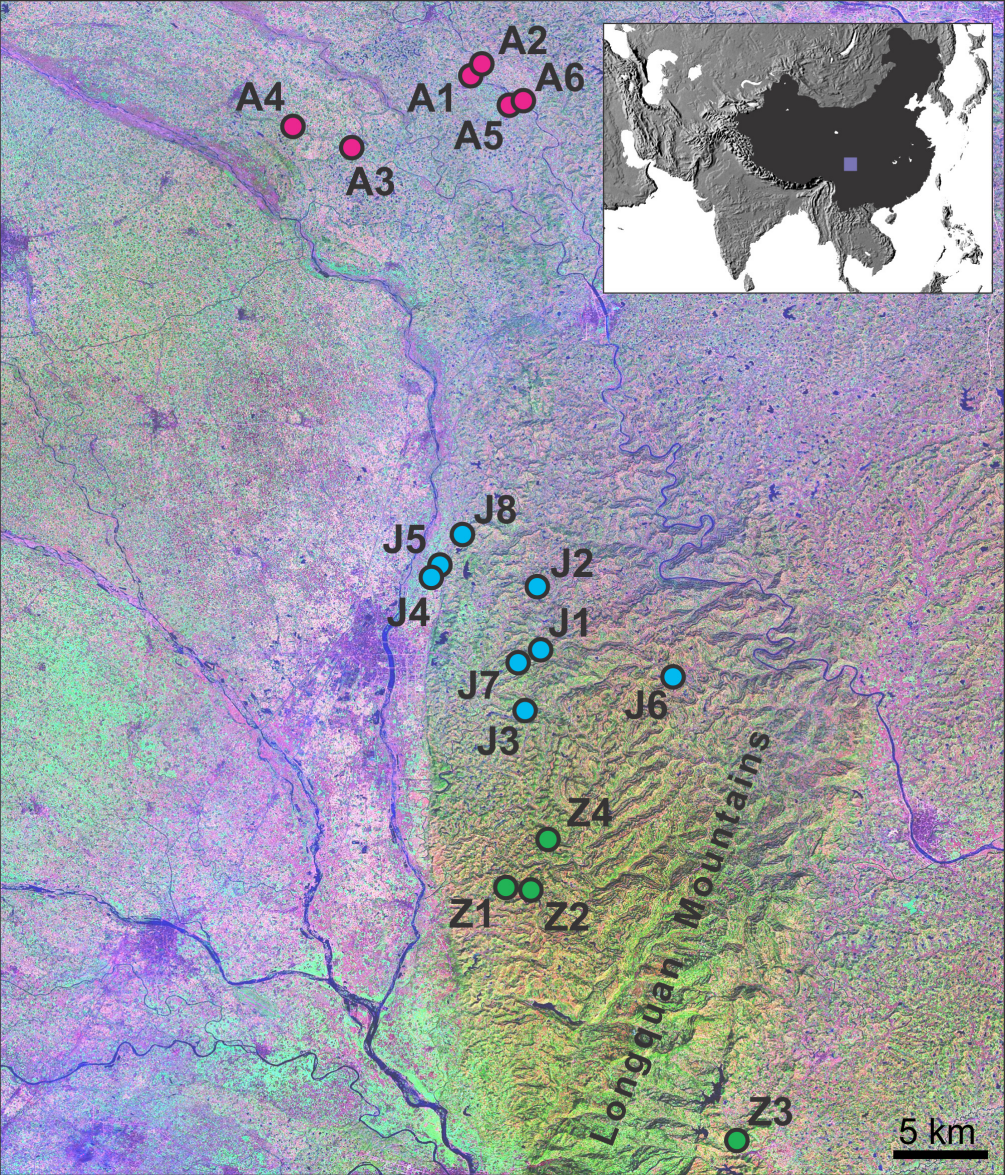
**Sampling sites of *Oncomelania h. robertsoni***. Sampling sites in the Deyang-Mianyang area, Sichuan Province, China. For site codes see Table 3 (A = Anxian County, J = Jingyang County, Z = Zhongjiang County). LandSat base map taken from MrSID (NASA). Note the Longquan Mountain Range (max. altitude > 1000 m) separating the southernmost site Z3 from the remaining sampling sites. The insert shows the location of the area in China.

This study focused on these aspects in a formerly endemic schistosomiasis area previously reported to harbour representatives of two distinct phylogroups [26] - the Deyang-Mianyang area in Sichuan Province (Figure 1).

Based on a combination of mtDNA and genome-wide ncDNA, we studied:

1) The phylogeography of the two *O. h. robertsoni *phylogroups, with an interest in understanding how these groups are distributed on a micro-scale, e.g., whether they occur in sympatry.

2) Regional and local population structures in space and time, focusing on the degree of population admixing as well as the potential correlation of population structure with physical barriers. Our working hypothesis is that strong habitat fragmentation in the study area, together with the long evolutionary history of the subspecies, causes a clear population structure reflecting the spatial structure of watersheds.

3) Patterns of dispersal under different isolation-by-distance scenarios to test whether isolation-by-distance can explain the phylogeographical patterns observed and if so, which dispersal scenario (i.e., straight line vs. waterway distances, etc.) is supported by genetic data. The hypothesis to be tested is that active or passive dispersal along waterways best explains the phylogeographical patterns observed.

Our study constitutes the first effort to combine mitochondrial and genome-wide nuclear data on the one hand, with phylogeographical, molecular clock, and phylogenetic analyses on the other hand, to unravel the microevolution of *Oncomelania h. robertsoni*. The findings of this study relate to the allopatric distribution of the two distinct snail phylogroups, the existence of presumably endemic lineages within the study area, the high degree of population admixing, the roles of large and small scale effects on population structure, and patterns of snail dispersal along watersheds, all of which have important parasitological implications. The results contribute to a better understanding of how population structure of the intermediate snail host affects susceptibility to schistosome infections, the means of snail, and thus parasite, dispersal, and phylogenetic tracking in *Schistosoma japonicum*.

## Results

### Phylogenetic and molecular clock analyses (COI data)

**Figure 2:**
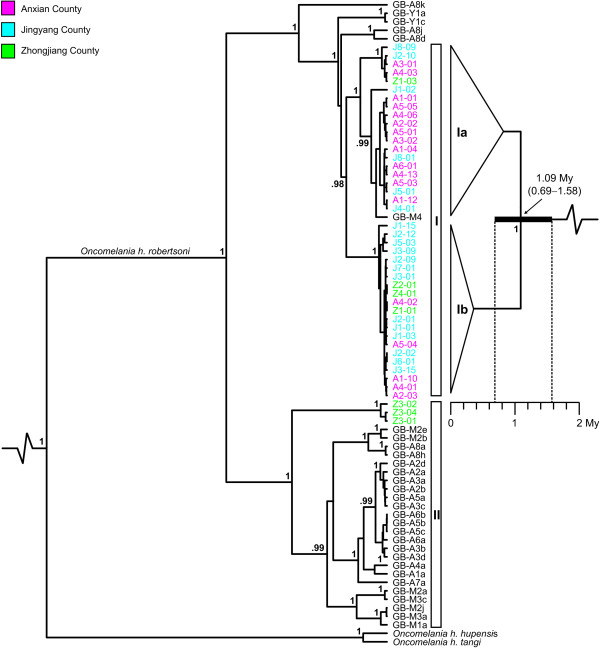
**COI phylogeny of *Oncomelania h. robertsoni***. Bayesian phylogenetic tree for *Oncomelania h. robertsoni *under the relaxed clock model inferred from the mitochondrial COI gene (left). The outgroup taxon, *O. minima*, was removed a posteriori. The two major phylogroups within *O. h. robertsoni *are labelled I and II. For specimen codes see Table 3 (ingroup sequences taken from GenBank are indicated by the prefix "GB"). A strict clock Bayesian tree of selected specimens of *O. h. robertsoni *from the Deyang-Mianyang area is shown on the right. Haplotypes from this area are color-coded according to counties (note that specimen GB-M4, previously studied by Attwood et al. [29], also originates from this area). For reasons of clarity, outgroups were removed posteriori and the overall topologies of the two distinct subclades Ia and Ib are indicated by triangles. The age of the MRCM of the two subclades and the respective 95% confidence interval are illustrated by a black bar. For both trees, all Bayesian posterior probabilities ≥ 0.95 are given next to the nodes.

The Bayesian phylogenetic analysis of specimens of *Oncomelania hupensis robertsoni *from throughout its distribution area under the relaxed-clock assumption resulted in the consensus tree shown in Figure 2 (left). The tree topology is very robust with all major and most medium-level clades being highly supported (i.e., Bayesian posterior probabilities [BPP] ≥ 0.95). In the tree, all *O. h. robertsoni *specimens form a monophyletic clade, clustering as sister group to *O. h. hupensis *(BPP = 1). Within *O. h. robertsoni*, two major phylogroups (BPP = 1) are evident. Phylogroup I (numbering according to Wilke et al. [26]) is primarily distributed in central and southern Sichuan as well as Yunnan. Specimens of phylogroup II occur mainly in western, but also in southern Sichuan.

All specimens from the northern Deyang-Mianyang area studied in the present paper cluster within phylogroup I, whereas all specimens from the southernmost population in the study area (Z3, see Figure 1) belong to phylogroup II. In our study area, these two phylogroups do not spatially overlap. However, overlapping is evident in southern Sichuan (see specimens GB-A8a, h vs. GB-A8d, j, k in Figure 1).

All specimens from the northern Deyang-Mianyang area (except for the extreme southern ones belonging to phylogroup II) form a monophyletic group (BPP = 0.98). Note that specimen GB-M4, previously studied by Attwood et al. [29], also clusters within this group as it comes from the same area. Within this monophyletic Deyang-Mianyang clade, two subclades (BPP = 1 each) are evident. Subclade Ia is highly diverse, comprising several deep lineages. Subclade Ib, however, is relatively shallow with fewer distinct groups. The two subclades do not show a clear geographical structure, with specimens from different groups occurring sympatrically.

The molecular clock analysis of the reduced data set, comprising phylogroup I specimens from the Deyang-Mianyang area (see strict clock Bayesian tree in Figure 2, right), indicates that the MRCA of the two subclades is approximately 1.09 My (95% CI: 0.69-1.58 My) old. Note that CI estimation includes both the error of branch length variation in the Bayesian analysis (i.e., 0.73-1.54 My) and the error of the clock rate for the HKY model (i.e., ± 0.22% My^-1^; see Methods section for details).

### Network analyses (COI and AFLP data)

**Figure 3:**
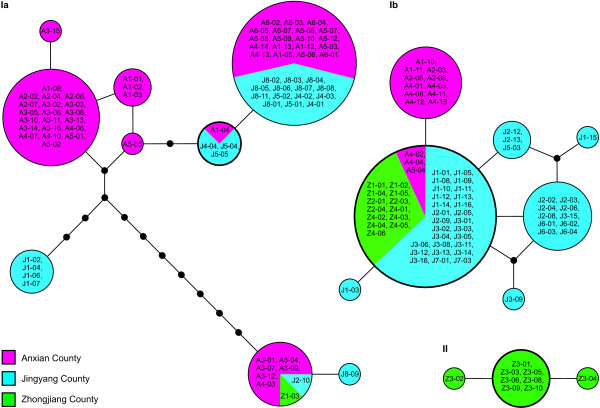
**COI network of *Oncomelania h. robertsoni***. Statistical parsimony haplotype networks for specimens from the Deyang-Mianyang area based on the COI gene (connection limit 95%). The three separate networks correspond to phylogroup II and the two subclades (Ia and Ib) of phylogroup I (see Figure 2). Haplotypes are color-coded according to counties. Areas of circles representing the haplotypes found are proportional to the number of specimens sharing the respective haplotype. Missing haplotypes are indicated by black dots. Haplotypes with the highest probability of being the ancestral haplotype in the individual networks are indicated by bold circles.

The COI-based TCS network (Figure 3) largely reflects the relationships seen in the phylogenetic trees presented above. The analysis resulted in three individual networks, which could not be connected by the statistical parsimony network algorithm in a parsimonious fashion (connection limit 95%).

The first network (Ia) consists of specimens from throughout the Deyang-Mianyang area (except for specimens from Z3) and corresponds to subclade Ia in the phylogenetic analysis (see Figure 2). The network consists of several distinct haplotypes with the putative ancestral haplotype (bold circle) being shared by only four specimens from three populations. In contrast, network two (Ib) shows a star-like structure with the putative ancestral haplotype being shared by 43 specimens from eight populations. This network corresponds to subclade Ib in Figure 2. The third network (labeled II) consists of nine specimens from the southernmost population Z3 (see Figure 1). Specimens belonging to this population are the only ones from the Deyang-Mianyang area studied that belong to phylogroup II (see Figure 2).

**Figure 4:**
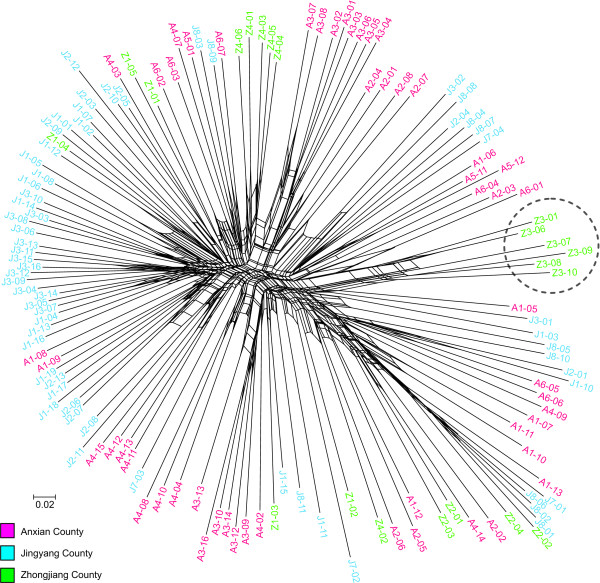
**AFLP network of *Oncomelania h. robertsoni***. NeighborNet network for specimens from the Deyang-Mianyang area based on AFLP data. The scale bar indicates ML distances. Specimen labels are color-coded according to counties. The distinct specimens from population Z3, which correspond to phylogroup II, are encircled.

The AFLP-based network generated in SplitsTree 4.10 (Figure 4) shows a star-like topology. Geographical structuring is weak, though specimens belonging to the same population tend to cluster together. This is particularly evident for the representatives of the southernmost population Z3 (see Figure 1; also see phylogroup II in Figure 2), which form the most distinct cluster in the network.

### AMOVA and SAMOVA analyses (COI and AFLP data)

A global AMOVA of populations by scaling order (SO) revealed for the COI data that about 73% of total molecular variation is distributed among, and only 27% within, populations (Table 1). For the AFLP data, the highest portion of genetic variation (87%) was found within populations and only 13% among populations. When using watersheds as scaling order (see Method section), the highest proportion of variance in the COI gene can be found at small to medium scale levels (70-75%).

**Table 1:** Results of AMOVAs in *Oncomelania h. robertsoni*

Source of variation	PP	WS 1	WS 2	WS 3	WS 4	WS 5	PG
COI							
V_a_	-	75.16***	71.72***	70.21***	64.65***	10.79***	86.31***
(among groups of populations)							
V_b_	72.94***	-1.99	2.33	5.39***	15.46***	63.54***	7.22***
(within groups of populations)							
V_c_	27.06***	26.83%**	25.94***	24.40***	19.88**	25.67	6.47
(within populations)							
AFLP							
V_a_	-	-27.48***	3.77***	5.66***	3.89***	1.57***	10.69***
(among groups of populations)							
V_b_	13.03***	40.45***	9.39***	8.18***	10.57***	12.11***	10.77***
(within groups of populations)							
V_c_	86.97***	87.03	86.84	86.16**	85.54*	86.32	78.54
(within populations)							

Partitioning of variation (V) for populations from the Deyang-Mianyang area derived from COI and AFLP data. Scaling orders are populations (PP), watersheds (WS), and phylogroups (PG); all values in %. Statistically significant results are marked with asterisks:* p ≤ 0.05, ** p ≤ 0.01, ***p ≤ 0.001.

For the AFLP data, the variation among watersheds is much smaller: the highest value of 6% was found at the medium scale level WS 3 (Table 1). However, the highest among-group variation in both the COI gene and AFLP data was detected when using the two distinct phylogroups (see Figure 2) as SO (86% and 11% in COI and AFLP data, respectively; see Table 1).

In contrast to the AMOVA, the spatial groupings suggested by the SAMOVA are less distinct. For the AFLP data, none of the F*_CT _*values (i.e., the variance among geographically adjacent groups relative to the total variance) is supported with p ≤ 0.1. For the COI gene, the highest F*_CT _*value (0.86) was obtained for a spatial clustering corresponding to the distribution of the two phylogroups. However, the respective p-value was only 0.058.

### Exact test and mismatch distribution (COI data)

Exact test and mismatch distribution analyses were carried out for specimens from the Deyang-Mianyang area belonging to the two distinct COI networks within phylogroup I (i.e., Ia and Ib in Figure 3). The exact test of sample differentiation based on haplotype frequencies rejected the global null hypothesis of panmixia for both subclades (p < 0.001). Pairwise analyses of individual populations indicated significant differences (p ≤ 0.05) in 90 out of 132 comparisons for subclade Ia and in 80 out of 144 comparisons for subclade Ib (raw data not shown here). Due to the rejection of panmixia, we could use mismatch distribution analyses only for testing the spatial extension model and not the demographic one (see Method section).

**Figure 5:**
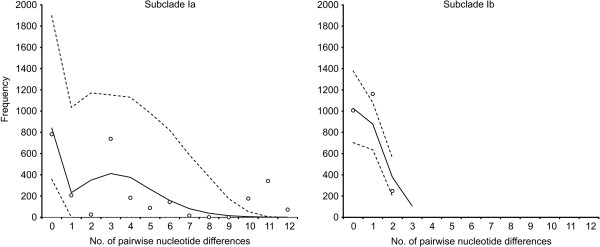
**COI mismatch distributions of *Oncomelania h. robertsoni***. COI-based mismatch distributions of specimens from the Deyang-Mianyang area belonging to subclades Ia and Ib (see Figure 2). The distributions of pairwise nucleotide differences (circles) were tested against the spatial expansion model (black line). The 95% confidence intervals of the coalescent simulations are indicated by dashed lines.

The individual mismatch distribution for subclade Ia shows a bimodal pattern with a maximal number of pairwise differences of 12 (Figure 5, left). Particular comparisons involving pairs with high nucleotide differences cluster outside the 95% confidence intervals of the coalescent simulations. Nonetheless, with a sum of squared deviation (SSD) value of 0.057 (p = 0.262), the spatial expansion model for this subclade is not rejected in favour of a demographic equilibrium. In contrast, the mismatch distribution for subclade Ib shows a relatively uniform distribution with the maximum number of pairwise differences being low (Figure 5, right). With a SSD value of 0.017 (p ≤ 0.001), the spatial expansion model for this subclade is rejected (note, however, that the variation in subclade Ib is very low, therefore, this result has to be treated with caution).

### Mantel tests (COI and AFLP data)

Mantel tests based on the COI gene showed a significant correlation between genetic and geographic distances for 10 out of 13 different geographic distance variables (Table 2). The best correlations were found for the following variables: Cost distance (*r_M _*= 0.59), total path (*r_M _*= 0.50), linear 1:10 and binary 1:10 (both *r_M _*= 0.49), as well as isotropic least-cost on-stream segments (*r_M _*= 0.48).

**Table 2:** Results of Mantel tests in *Oncomelania h. robertsoni*

Geographic distance variable	*r_M _*(COI)	*r_M _*(AFLP)
Euclidean distance	0.454***	0.217
*Isotropic least-cost distance*		
Cost distance	0.588***	0.249*
Proportions	0.103	0.126**
Onstream segments	0.480***	0.240*
Total path	0.495***	0.249*
*Anisotropic least-cost distance*		
Linear 1:10	0.490***	0.297*
Linear 1:10 proportion	0.115	0.054
Binary 1:10	0.490***	0.246*
Binary 1:10 proportion	0.129	0.166**
Linear 1:100	0.350***	0.131
Linear 1:100 proportion	0.141**	0.228***
Binary 1:100	0.396***	0.133
Binary 1:100 proportion	0.132**	0.264***

Indices for correlation of genetic (COI and AFLP data) distances and geographic distances in *Oncomelania h. robertsoni *from the Deyang-Mianyang area (*r_M_*). Statistically significant results are marked with asterisks: * p ≤ 0.05, ** p ≤ 0.01, *** p ≤ 0.001

The Mantel tests for the AFLP data showed very similar results. Here, 9 out of 13 different geographic distance variables showed significant correlations with genetic distance (Table 2). Moreover, the five best values were obtained for the very same variables as in the COI gene with values of *r_M _*= 0.25, 0.25, 0.30, 0.25 and 0.24, respectively. The distance variable with the overall best correlation in both data sets was cost distance.

The Mantel test performed on genetic distances calculated for the COI and AFLP data sets also showed a significant relationship between these two different sets of genetic data (*r_M _*= 0.486, p = 0.048).

## Discussion

### Phylogeny of *Oncomelania h. robertsoni*

From the present study it becomes evident that the two deviant phylogroups in *O. h. robertsoni *have no clear spatial structure. Though phylogroup II appears to have a more southern and eastern distribution [26], ranges are adjacent (e.g. in the southern part of our study area) or even overlap as previously shown for an area in southern Sichuan [26].

**Figure 6:**
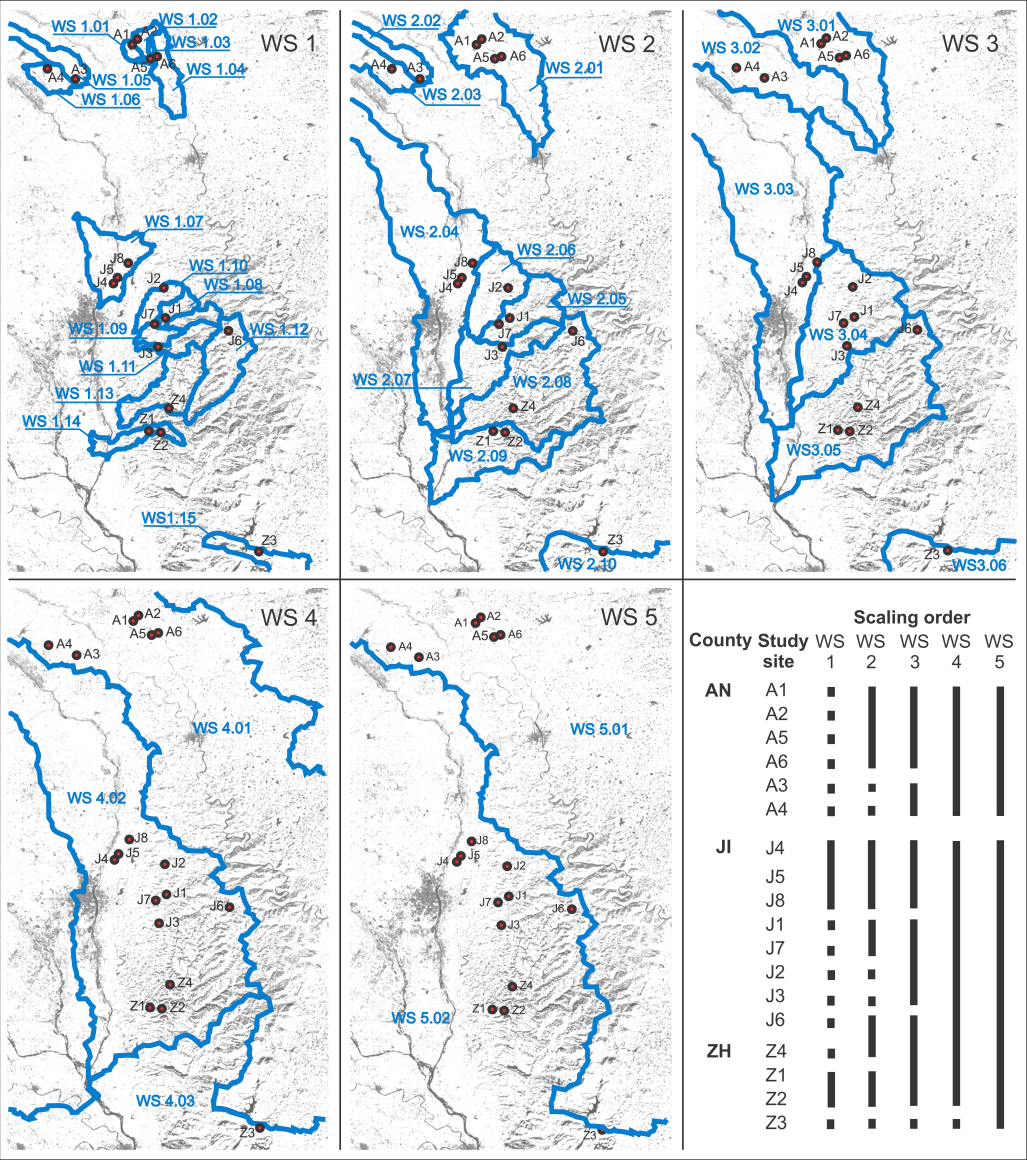
**Classification of watersheds in the Deyang-Mianyang area**. Watershed (WS1-5) classification of 18 study sites of *Oncomelania h. robertsoni*. For county and site codes see Table 3.

Interestingly, the disjunctive distribution of the two phylogroups in the Deyang-Mianyang area cannot be explained with watershed classifications (Figure 6) or other limnological parameters. Instead, the two groups remain separated by the largest mountain range in the area, the Longquan Mountains, reaching altitudes of > 1000 m (Figure 1). This geographical barrier might be the reason why phylogroup II specimens have not (yet) reached the northern Deyang-Mianyang area, while we did find a sympatric population in southern Sichuan (see GB-A8a, h vs. GB-A8d, j, k in Figure 1).

The observed geographical patterns could be the result of increasing secondary contact of phylogroups I and II. However, whether time of isolation has been long enough to allow for the development of pre- or postzygotic barriers to speciation [30] remains unclear as the age of the MRCA of phylogroups I and II could not be estimated due to the rejection of the strict molecular clock model in the full data set.

Nevertheless, given the topology of the phylogenetic tree (Figure 2), this MRCA must be much older than 0.69-1.58 My, possibly dating back to the early Pliocene or even late Miocene.

The generally patchy distribution of the phylogenetically old phylogroups I and II throughout Yunnan and Sichuan might be largely due to the highly fragmented and often isolated habitats of *O. h. robertsoni *in the hilly and mountainous regions of western China [31,32]. In this regard, *O. h. robertsoni *differs considerably from its eastern Chinese sister taxon, *O. h. hupensis*. Being distributed mainly within and along the floodplains of the Yangtze River, the latter taxon is strongly affected by annual flooding [16,22], leading to extensive gene flow and population admixing [19,33].

Unfortunately, the two phylogroups are not as clearly separated in the AFLP data set compared to the mtDNA data, further complicating decisions as to the taxonomic status of these two groups. While phylogroup II specimens form a distinct cluster in the AFLP-based network (Figure 4) and show a significant grouping in the AMOVA analyses (Table 1), overall divergence remains low. Possible explanations include the exchange of genes and the differential performance of AFLP. While this method works well for closely related genotypes, unrelated genotypes may contribute considerable noise to the data set [34]. This is due to the fact that AFLP only uses fragment length as a criterion and not the actual DNA sequences. Accordingly, co-migrating fragments (i.e., those of the same length) are considered to be homologous. This assumption, however, is an oversimplification [35] and the fraction of non-identical co-migrating (i.e., homoplasious) fragments largely depends on phylogenetic distance. Values can range from approximately 10% in closely related genotypes to as much as 100% in distantly related taxa [34]. Given these findings, it appears possible that the lack of a clear differentiation of the two ancient phylogroups based on AFLP data is due to noise problems (also see Caballero & Quesada [36]). However, at a lower taxonomic level (i.e., within phylogroups), this problem seems to be less severe as indicated by, for example, the tendency of specimens originating from the same population to cluster together in the network analysis (Figure 4) and the results of the Mantel test showing concordance of COI and AFLP data.

No matter whether the two *O. h. robertsoni *phylogroups represent distinct species or not, implications for understanding disease transmission might be substantial due to the close co-evolutionary relationships between *S. japonicum *and its hosts, both intermediate and definitive. Not only are there distinct parasite strains that correlate to snail subspecies [10-12], but also to definitive host species (i.e., buffalo, cattle and humans vs. goats, pigs, dogs and cats [13,37]; for a review of host species see McManus et al. [38]). These data indicate a distinct hierarchical genetic structure in *S. japonicum*: 1) genotype groups according to intermediate snail host taxa, 2) subgroups corresponding to definitive hosts, and 3) possibly MLGs within strains.

This presumably high degree of host specificity calls for a closer investigation of the parasites within the respective *O. h. robertsoni *phylogroups. Of interest is also whether snails belonging to different phylogroups show different rates of susceptibility to infection or even different scales of resistance ("Red Queen" [39]). Finally, cases of schistosomiasis re-emergence in Sichuan and Yunnan provinces should be studied in respect to snail phylogroups (i.e., phylogroup admixing and/or replacement).

### Regional and local population structures in space and time

Our phylogenetic analyses show that specimens of all but the southernmost population from the Deyang-Mianyang area (Z3) belong to phylogroup I. Moreover, these specimens form a relatively old and well supported monophyletic group in our analyses (Figure 2), indicating distinct geographical pattern at a regional scale and corroborating the status of the area as being historically endemic for schistosomiasis. Within this clade (i.e., within the Deyang-Mianyang area), the geographical structure appears to be weaker. Nonetheless, the results of the AMOVAs show significant partitioning of genetic variance according to watersheds (Table 1), both for the COI and, to a lesser, extent, the AFLP data sets. In addition, local demographic processes appear to act heavily on individual populations, causing medium (COI gene) to high (AFLP data) partitioning of variation within populations (Table 1).

Interestingly, the Deyang-Mianyang clade consists of two well supported subclades (see subclades Ia and Ib in Figure 2). These two groups do not appear to be spatially structured as specimens belonging to both groups occur in sympatry. However, individual mismatch analyses of subclade Ia and Ib specimens reveal contrasting patterns in terms of ruggedness and number of maximum pairwise nucleotide differences (Figure 5). Acknowledging that mismatch patterns are difficult to interpret [40], the pattern seen in Ia might suggests the existence of old and diverse lineages and possibly indicates a relatively stable population structure (also see Figure 3). In contrast, the pattern seen in Ib points to young lineages with no sudden spatial expansions being detectable. The most parsimonious explanation for these striking differences in age, structure and spatial history between the two subclades is that both groups were previously isolated and only recently came in contact. In fact, our genetic data indicate a long and diverse evolutionary history of *O. h. robertsoni *populations in the Deyang-Mianyang area starting some 0.69-1.58 My ago (see Figure 2).

Overall, the combined results of our phylogenetic and phylogeographical analyses indicate, for the first time, a hierarchical population structure in *O. h. robertsoni*, caused by small to medium scale factors. Scales of concern are: 1) the population level characterized by local demographic processes, 2) the watershed level with watershed boundaries acting against gene flow to different degrees, and 3) the regional level of a formerly endemic schistosomiasis area, supporting a high degree of endemic snail lineages. In addition, we see large scale effects such as increasing secondary phylogroup contact, possibly caused by long-range dispersal of *O. h. robertsoni *throughout Sichuan and Yunnan provinces.

Of these scale effects, watershed effects are important but do not appear to be the dominant factor. Therefore, our null hypothesis that the strong habitat fragmentation in the study area and the long evolutionary history of the subspecies are best reflected in a population structure corresponding to watershed distribution, has to be rejected.

Instead, larger scale effects together with considerable population admixing might also contribute to the genetic patterns observed. Using the words of Ian Flemings's fictional British Secret Service agent James Bond as a metaphor, which were first introduced in the 1956 novel "*Diamonds are Forever*", the population structure of *O. h. robertsoni *within this formerly endemic schistosomiasis area is stirred, but not shaken.

### Patterns of dispersal under different isolation-by-distance scenarios

For both data sets AFLP and COI, we see clear isolation-by-distance effects, that is, a significant correlation between geographic and genetic distance. This is a rather unexpected finding as, to our knowledge, such an effect has not been shown before for a currently or historically endemic schistosomiasis area in China. Though this might be partly due to the fact that only few detailed regional studies exist, previous studies mainly suggested either large scale admixing effects caused by long-range dispersal [26] or strong fragmentation effects at the landscape level [25,32] as the prevailing biogeographical processes in *O. h. robertsoni*.

Moreover, both marker systems used, although being very different in terms of performance and target molecules, indicate the very same distance parameters as those having the highest effect (see Table 2). Though these effects are relatively small in the AFLP data set, three isotropic least-cost and two anisotropic least-cost distance variables show the highest *r_M _*values. In contrast, Euclidean distance (i.e., simple straight-line distance) shows a weaker influence in both data sets, and is non-significant in the AFLP dataset.

Acknowledging that the biology and ecology of *O. h. robertsoni *are still not fully understood, these findings argue for a primary dispersal along waterways. This could be caused either passively by currents or flooding, attachment of eggs to aquatic or amphibious animals, or human-mediated [16,39,41]. It could also be actively as *O. h. robertsoni *is known to be negatively rheotropic [39].

Water birds and other vectors not bound to waterways likely play only a minor role for the regional dispersal of *O. h. robertsoni*. This, in turn, means that our null hypothesis (active or passive dispersal along watersheds best explains the phylogeographical patterns observed) cannot be rejected. The implications for understanding disease transmission, however, might be considerable. This is because our findings now allow for incorporating genetically-derived snail data in models of disease transmission. Acknowledging that further studies are necessary for a better understanding of dispersal pathways in space and time, we suggest using an anisotropic cost distance measure of *O. h. robertsoni *in future analyses, including network-based transmission models [42].

## Conclusions

Our multi-locus genetic analyses of the *Schistosoma japonicum *snail host *Oncomelania hupensis robertsoni *in a formerly endemic schistosomiasis area revealed a deep, complex and hierarchical structure, likely reflecting a long and diverse evolutionary history. It is characterized by population, watershed, regional and large scale effects acting together to different degrees. Despite the strong admixing of genotypes at various spatial scales, we were able to identify local processes affecting distribution patterns. Our data indicate that small and especially medium-level watershed scaling orders well reflect the observed population structures, with clear isolation-by-distance effects along waterways being evident. In addition, all snail lineages within our study area, i.e., at the regional level, appear to be endemic, supporting the concept of endemic schistosomiasis areas. Finally, large scale effects are evident (e.g., the presence of phylogenetic subclades), likely reflecting secondary contact of previously isolated lineages. In fact, our study raises the question about the existence of two distinct and partly sympatric *Oncomelania *species in western China. At least from a strictly phylogenetic standpoint, such a possibility can no longer be dismissed outright.

Finally, there are several important implications for understanding disease transmission arising from our study:

1) From a co-evolutionary standpoint, the existence of two distinct phylogroups in *O. h. robertsoni *argues for future studies relative to the distinctness of the respective parasites.

2) The admixing of previously isolated regional groups (here subclades Ia and Ib) calls for testing for increased rates of susceptibility to infection in these populations. 3) The high level of snail lineage endemicity within a formerly endemic schistosomiasis area warrants future geospatial analyses for a better understanding of the boundaries of transmission areas.

4) Most, but not all, snail populations are genetically highly diverse, raising questions about the effect of mollusciciding campaigns on snail population structure, and thus susceptibility to infection.

5) Local snail dispersal mainly occurs along waterways and can be best described by using cost distance, thus potentially enabling a more precise modelling of snail, and therefore parasite, dispersal.

While our study demonstrates the usefulness of phylogeographical analyses for reconstructing medium- and long-term evolutionary histories of snail host populations, it also calls for future longitudinal studies to better clarify short-term dynamics. These studies should also be based on larger sampling sizes per population to enable sophisticated population genetic analyses for better addressing local snail dynamics and the impact of demographical effects.

Moreover, as *O. h. robertsoni *shows a high degree of diversity and as the fraction of noise in AFLP data sets increases with genetic distance, we suggest that future larger-scale studies utilize additional markers, such as microsatellites, in order to better unravel the deeper, "stirred" structures in this taxon.

## Methods

### Study area and field work

The Deyang-Mianyang area is located in the northwestern part of the Sichuan Basin (Sichuan Province). Being separated from the Tibetan Plateau by the Longmenshan Fault, the area is characterized by a series of low hills and alluvial plains. Several major rivers flow into the Yangtze River, which passes through the southern part of Sichuan Basin.

Our sampling design comprised 18 sites located in Anxian, Jinyang and Zhongjiang counties (Figure 1, Table 3). The largest straight-line distance between sites is approximately 63 km. Amphibious *Oncomelania hupensis robertsoni *specimens in each site were hand-collected in April 2008 from vegetation along small irrigation channels (< 3 m across at bank flow) and immediately preserved in 80% ethanol. Strictly for semantic reasons, specimens coming from the same site are here referred to as a population.

**Table 3:** Locality and marker information for *Oncomelania h. robertsoni*

County	Study site	Latitude	Longitude	Elevation	# Specimens studied	
(county code)	(site code)	(in °N)	(in °E)	(in m)	AFLP	COI
Sichuan Province: Deyang-Mianyang area						
Anxian (AN)	Baiguo 01 (A1)	31.43616	104.44606	540	9	10
Anxian (AN)	Baiguo 02 (A2)	31.42958	104.44214	534	8	7
Anxian (AN)	Huangta 03 (A3)	31.39258	104.37942	557	14	16
Anxian (AN)	Jinguang 10 (A4)	31.40378	104.34948	567	12	14
Anxian (AN)	Jingquan 01 (A5)	31.41634	104.46281	531	3	11
Anxian (AN)	Jingquan 03 (A6)	31.41706	104.46676	532	7	7
Jingyang (JI)	Dashu 04 (J1)	31.13285	104.47710	560	18	16
Jingyang (JI)	Dongchao 05 (J2)	31.16546	104.47636	533	12	11
Jingyang (JI)	Gaohuai 07 (J3)	31.10173	104.46974	505	16	14
Jingyang (JI)	Gongqiao 02 (J4)	31.17130	104.42275	500	-	4
Jingyang (JI)	Gongqiao 03 (J5)	31.17529	104.42538	505	-	5
Jingyang (JI)	Longju 03 (J6)	31.11858	104.54519	621	-	4
Jingyang (JI)	Shiban 01 (J7)	31.12595	104.46544	536	4	2
Jingyang (JI)	Xinquan 03 (J8)	31.19181	104.43809	509	11	10
Zhongjiang (ZH)	Xindong 03 (Z1)	31.01038	104.45952	498	5	5
Zhongjiang (ZH)	Xindong 06 (Z2)	31.00835	104.47175	538	4	3
Zhongjiang (ZH)	Xinglong 05 (Z3)	30.87877	104.57958	495	6	9
Zhongjiang (ZH)	Xinzheng 04 (Z4)	31.03464	104.48192	517	6	6
Mianzhu (MH)	Mianzhu (GB-M4)**	30.067	104.138		-	1
Sichuan Province: other sites						
Xichang	Gucheng (GB-A1)*	27.93525	102.20540		-	4
Xichang	Gucheng (GB-A2)*	27.93180	102.19620		-	4
Xichang	Minhe (GB-A3)*	27.87505	102.30867		-	4
Xichang	Zhoutun (GB-A4)*	27.80000	102.20400		-	4
Xichang	Gucheng (GB-A5)*	27.79950	102.30870		-	4
Xichang	Gucheng (GB-A6)*	27.79730	102.31570		-	4
Xichang	Jingjiu (GB-A7)*	27.74680	102.19030		-	4
Miyi	Shuanggou (GB-A8)*	26.96370	102.13280		-	12
Danling	Xiaoqiao (GB-M1)*	29.99163	103.41580		-	2
Dongpo	Magau (GB-M2)*	30.13993	103.61167		-	10
Meishan	Zhongfu (GB-M3)*	30.03730	103.90020		-	5
Yunnan Province						
Dali	Zi Ran (GB-Y1)*	25.45100	100.20070		-	8

Populations collected and genetic markers studied in *Oncomelania h. robertsoni *from Sichuan and Yunnan provinces. Previously studied populations are marked with asterisks: *Wilke et al. [26], **Attwood et al. [29].

For assessing local phylogroup distribution (see Goal 1), we also included in our study additional specimens of *O. h. robertsoni *from Sichuan and Yunnan provinces (Table 3) for comparison. These specimens, previously studied by Wilke et al. [26], largely cover both the distribution area of the subspecies and its overall genetic variation.

### Watershed classification and calculation of geographical distances among populations

For analyses of genetic structure and for assessing the role of physical barriers (see Goal 2), a hierarchical watershed assignment was inferred from a digital elevation model (DEM) using Spatial Analyst in ArcGIS 9.2 (ESRI), with scaling orders being determined using Strahler stream ordering (Figure 6) [43]. Study sites were classified according to watershed membership based on five scales of watersheds, with the largest based on the highest Strahler stream order (stream order 9) and the smallest based on the lowest stream order in which changes in watershed classification were observed (stream order 4, for which almost all sites resided in their own watershed) (Figure 6).

Geographical distance variables among study sites were calculated for testing patterns of snail dispersal under different isolation-by-distance scenarios (see Goal 3). Distance scenarios include A) simple straight-line Euclidean distances, B) least-cost distances, (isotropic) and C) least-cost distances taking vertical distance and direction into account (anisotropic) (see Table 4). All Euclidean and cost distances were calculated using Spatial Analyst in ArcGIS 9.2. Euclidean distance was the simple straight-line distance between each pair of study sites, not including elevation. Straight-line distance is known to ignore the influence of heterogeneous landscape and topographic factors [42], thus alternative distance measures were generated to test the influence of streams and topology on snail dispersal. The isotropic least-cost distances were based on on-stream versus off-stream movement, with the least-cost distance model favouring on-stream movement over off-stream movement by an order of magnitude penalty for each off-stream distance unit. To incorporate the influence of uphill/downhill and upstream/downstream movements, on-stream versus off-stream weights and vertical weights were combined into four alternative models, which we refer to as anisotropic owing to their directional dependence related to slope. The four models used binary or linear vertical response functions, and two weighting approaches (Table 4). For binary anisotropic models, all positive slopes (uphill) were weighted as more costly, by a factor of ten, than negative slopes (downhill). For linear anisotropic models, the most extreme uphill slope in the DEM was weighted as ten times as costly as the steepest downhill slope, transitioning linearly between the two values [44]. Off-stream versus on-stream movement in these models was weighted either as described above for the isotropic model (one order of magnitude weighting difference: "10:1"), or two orders of magnitude ("100:1"). Moreover, two different measures of distance were used for all isotropic and anisotropic model variants, the cost distance and the proportion of the least-cost path that consisted of onstream segments, the latter emphasizing the importance of paths dominated by stream cells (Table 4). Finally, for two other isotropic distance models were explored: the cumulative distance of on-stream segments, and Euclidean distance along the least-cost path.

**Table 4:** Distance models applied

	Components		
Distance model	Off-stream weight*	Slope	Model description
*Euclidean distance*			
Euclidean distance	1	N/A	Straight-line distance
*Isotropic least-cost distance*			
Cost distance	10	N/A	Isotropic least-cost path distance
Proportions	10	N/A	Proportion of the path that consists of on-stream segments
Onstream segments	10	N/A	Cumulative distance of on-stream segments along least-cost path
Total path	10	N/A	Cumulative, unweighted distance along least-cost path
*Anisotropic least-cost distance*			
Linear 1:10	10	Linear increase; 1 at most negative slope, 10 at most positive slope	Anisotropic least-cost path distance
Linear 1:10 proportion	10	Linear increase; 1 at most negative slope, 10 at most positive slope	Proportion of on-stream segments
Binary 1:10	10	Negative slope: 1 Positive slope: 10	Anisotropic least-cost path distance
Binary 1:10 proportion	10	Negative slope: 1 Positive slope: 10	Proportion of on-stream segments
Linear 1:100	100	Linear increase; 1 at most negative slope, 10 at most positive slope	Anisotropic least-cost path distance
Linear 1:100 proportion	100	Linear increase; 1 at most negative slope, 10 at most positive slope	Proportion of on-stream segments
Binary 1:100	100	Negative slope: 1 Positive slope: 10	Anisotropic least-cost path distance
Binary 1:100 proportion	100	Negative slope: 1 Positive slope: 10	Proportion of on-stream segments

Alternative geographic distance variables estimated between all pairs of sampling sites. *Relative to on-stream segments.

### DNA extraction

Genomic DNA was extracted from 207 ethanol preserved specimens using parts of the foot muscle or whole animals. For details of the CTAB isolation protocol used see Wilke et al. [26]. Final isolation products were diluted in 40 μl of purified water, and quantity and quality of DNA determined using a Thermo Scientific Nanodrop 2000 micro-volume UV-VIS spectrophotometer.

DNA vouchers are stored at -80°C in the University of Giessen Systematics and Biodiversity collection (UGSB). Note that individual specimens typically had to be destroyed during DNA isolation. Therefore, high-resolution images of the respective specimens were generated using the digital microscope Keyence VHX-600, and deposited in the UGSB database.

### PCR and DNA sequencing

To explore intraspecific diversity and evolutionary relationships among populations, a fragment of the cytochrome *c *oxidase subunit I (COI) gene was sequenced. Forward and reverse primers for PCR amplification and DNA sequencing were LCO1490 [45] and COR722b [46]. Bidirectional determination of sequences was performed either on a Long Read IR^2 ^4200 sequencer (LI-COR, Lincoln, NE, USA) using the Thermo Sequenase Fluorescent Labeled Primer Cycle Sequencing kit (Amersham Pharmacia Biotech, Piscataway, NJ, USA) or on a ABI 3730 XL sequencer (Life Technologies, Carlsbad, CA, USA) using the Big Dye Terminator Kit (Life Technologies, Carlsbad, CA, USA). Forward and reverse sequences were then aligned and trimmed in BioEdit 7.0.9 [47], resulting in a 638 bp long overlapping fragment of the COI gene.

All newly generated sequences for specimens from the Deyang-Mianyang area were deposited at GenBank (JN818846-JN818999). For accession numbers of the remaining ingroup sequences (prefix "GB") see Wilke et al. [26].

### AFLP genotyping

For micro-scale analyses of the genetic structure of *O. h. robertsoni *within the Deyang-Mianyang area, genome-wide ncDNA was studied applying the AFLP method with the protocol being slightly modified from the one suggested by Vos et al. [48]. Major steps are as follows:

1) Digestion and ligation were performed simultaneously in a total reaction volume of 10 μl containing ddH_2_O, 100X bovine serum albumin, 10X reaction buffer, 0.2 μl of 5 μM EcoRI and MseI adapter each, *MseI *(4 u/μl) and 0.16 μl *EcoRI *enzyme (20 u/μl), 400 u/μl T4 ligase, and 1 μl genomic DNA. The mixture was incubated for 12 hours at 37°C and products were checked on 1% agarose gels.

2) Ligated products were diluted 1:40 and an initial selective amplification step was performed in a 13 μl reaction mixture containing ddH_2_O, 1.3 μl ThermoPol buffer (10X), 1.5 μl dNTPs (2.0 mM each), 0.1 μl pre-selective primers *EcoR*I (+1) and *Mse*I (+1 or +2 selective bases) (each 75 ng/μl), 0.2 μl MgCl (2.5 μM), 0.1 μl taq polymerase (5 u/μl), and 1 μl ligated product. Cycling conditions comprised 2 min at 50°C, 3 min at 94°C, 30 cycles with each 30 sec at 94°C, 1 min at 56°C and 1 min at 72°C, and a final elongation step of 7 min at 72°C.

3) Final selective amplification was conducted in a 6 μl reaction mix containing ddH_2_O, 0.6 μl ThermoPol buffer (10X), 0.6 μl dNTPs (2.0 mM each), 0.06 μl taq polymerase (5 u/μl), 0.10 μl and 0.18 μl of 700 nm and 800 nm fluorescent labeled *EcoR*I primers (1 μM), respectively, and 0.20 μl *Mse*I primers (10 μM, +3 or +4). Exact sets of primer pairs (with only selective bases indicated) were: *EcoR*I-AAC/MseI-ACTA, *EcoR*I-AAC/*Mse*I-ACAT, *EcoR*I-AAC/*Mse*I-CAC, *EcoR*I-AAC/*Mse*I-CAT, *EcoR*I-AGC/*Mse*I-ACAT, *EcoR*I-AGC/*Mse*I-CAC, *EcoR*I-AGC/*Mse*I-CAT and *EcoR*I-AGC/*Mse*I-CGT (also see Table 5). In preliminary screening and reproducibility studies of 20 primers sets, these combinations were found to generate clear AFLP profiles. Primer combinations with low reproducibility and/or producing fuzzy bands were discarded. Cycling reactions comprised 10 cycles of 30 sec at 94°C, 30 sec at 65°C, and 1 min at 72°C, followed by 25 cycles of 30 sec at 94°C, 30 sec at 55°C, and 1 min at 72°C. Final extension lasted 2 min at 72°C.

**Table 5:** Information on AFLP

Primer name	Primer sequence
Adapter	
*EcoR*I Forward	5'-CTC GTA GAC TGC GTA CC-3'
*EcoR*I Reverse	5'-AAT TGG TAC GCA GTC TAC-3'
*Mse*I Forward	5'-GAC GAT GAG TCC TGA G-3'
*Mse*I Reverse	5'-TAC TCA GGA CTC AT-3'
Pre-selective primers	
*EcoR*I-A	5'-GAC TGC GTA CCA ATT CA-3'
*Mse*I-C	5'-GAT GAG TCC TGA GTA AC-3'
*Mse*I-AC	5'-GAT GAG TCC TGA GTA AA C-3'
Selective primers	
IRD-800 *EcoR*I-AAC	5'-GAC TGC GTA CCA ATT CAA C-3'
IRD-700 *EcoR*I-AGC	5'-GAC TGC GTA CCA ATT CAG C-3'
*Mse*I-CAC	5'-GAT GAG TCC TGA GTA ACA C-3'
*Mse*I-CAT	5'-GAT GAG TCC TGA GTA ACA T-3'
*Mse*I-CGT	5'-GAT GAG TCC TGA GTA ACG T-3'
*MseI*-ACTA	5'-GAT GAG TCC TGA GTA AAC TA-3'
*MseI*-ACAT	5'-GAT GAG TCC TGA GTA AAC AT-3'

Adapters and PCR primer sequences used for AFLP genotyping of *Oncomelania h. robertsoni*.

Selective amplified fragments were separated on a 6.5% LI-COR KB^PLUS ^denaturing polyacrylamide gel utilizing a LI-COR LongRead IR^2 ^4200 sequencer. AFLP bands and respective sizing standard (LI-COR Biosciences) were digitally captured with the e-Seq v. 2.0 software (LI-COR Biosciences).

Fragment scoring of AFLP profiles for 135 specimens of *O. h. robertsoni *was performed with the Saga^Mx ^module (LI-COR Biosciences), with bands recognized as present (1) or absent (0).

To obtain correctly sized bands, to minimize the effects of homoplasious fragments (see Discussion), and to further increase reproducibility, only bands ranging from 65-375 bp in length and being present in at least two samples were recognized and compared to the sizing standard [49]. Using eight primer combinations, a total of 237 highly polymorphic bands (ranging from 22-40 bands per combination) were screened.

### Phylogenetic and molecular clock analyses (COI gene)

The phylogenetic data set consisted of 154 specimens from the Deyang-Mianyang area, additional 66 specimens from Sichuan und Yunnan provinces as well as the 3 outgroup taxa (*O. minima *[GenBank: DQ212795], *O. h. hupensis *[GenBank: AF254488] and *O. h. tangi *[GenBank: DQ212796]).

To avoid a bias of model selection and subsequent phylogenetic analyses towards population-level relationships, we reduced our data set of 220 *O. h. robertsoni *sequences to a subset of 74 ingroup sequences, representing all populations and most haplotypes.

From this "full" data set, we inferred the best-fit model of sequence evolution based on the Bayesian information criterion by conducting dynamical likelihood ratio tests in jMODELTEST 0.1.1 [50].

We then ran two phylogenetic analyses in order to test whether the molecular clock hypothesis is accepted (i.e., whether a strict molecular clock can be assumed). As model-choice criterion, we used the Bayes factor (BF) [51]), that is, the ratio of the marginal likelihoods of two given models. If the models differ by a factor of 2 ln(BF) > 6, then there is strong evidence against the null model [52]. The marginal likelihood can be approximated by using the harmonic mean of the likelihood values from a MCMC sample. An advantage of the Bayes factor compared to likelihood ratio tests is that it allows for comparison of non-nested models [52]. In order to generate the Bayes factor, we conducted Bayesian Inference analyses (strict clock vs. relaxed clock assumption) in BEAST v1.6.1 [53]. The nucleotide substitution model to be applied was the one obtained from jMODELTEST (i.e., the HKY+I+Γ model) and the clock model either a strict clock or a relaxed clock with an uncorrelated exponential distribution. MCMC progress was monitored with TRACER 1.5.0 [53], and the analysis was terminated after stationarity of chain likelihood values was achieved (i.e., after 10.000.000 generations). The posterior output of each of the models was then used to estimate the Bayes factor in order to decide whether a strict clock can be assumed. With harmonic means of -ln = 2865.2 and -ln = 2828.4 for the strict clock and relaxed clock models, respectively (inferred with TRACER), and a Bayes factor of 73.6, there is strong evidence against the strict clock model. Therefore, the relaxed-clock model was applied to subsequent analyses of the full data set.

In order to achieve an optimal reconstruction of phylogroup distribution within the Deyang-Mianyang area (see Goal 1), we generated a second ("reduced") dataset by keeping only Deyang-Mianyang specimens as ingroups and one specimen each of *O. minima *and *O. h. hupensis *as outgroups. This data set was subjected to the same analyses as the full data set. Based on the best fit HKY model, the harmonic means of the strict clock and relaxed clock models were -ln = 1800.4 and -ln = 1800.0, respectively. The resulting Bayes factor of 0.8 indicated no evidence against the strict clock model. We therefore considered this reduced data set suitable for molecular clock analyses under a strict clock assumption.

For estimating the age of the most recent common ancestor (MRCA) of the Deyang-Mianyang specimens (see Goal 2), we used the molecular clock approach suggested by Wilke et al. [54]. In the absence of an *Oncomelania*-specific molecular clock rate, we utilized the trait-specific COI Protostomia rate of 1.24% ± 0.22% My^-1 ^for the HKY model [54]. This external clock rate, applicable to strict clock models only, has been shown to be valid in protostomian taxa with a similar biology and life-history (i.e., dioecious tropical or subtropical taxa with a generation time of approximately one year and a body size of approximately 2-50 mm). All these conditions are met by *O. h. robertsoni*. We then calculated the 95% confidence interval for our time estimate incorporating both the error of the phylogenetic analysis (i.e., the node-depth variation of individual trees calculated in BEAST) and the error of the clock rate (see above) by utilizing a propagation of uncertainty approach [54].

Note that we did not correct our clock estimates for ancestral polymorphism since the trait-specific Protostomia COI clock applied here is also not corrected [54]. As this external clock is based on phylogenetic events that are, on average, 3 My old, there might be a small bias towards overestimation of divergence times for events younger than 3 My and towards underestimation for events older than that.

### Phylogeographical analyses (COI gene)

For phylogeographical analyses (both network analysis and test for genetic structure) of *O. h. robertsoni*, we used COI sequences from a total of 154 specimens (here called the "Deyang-Mianyang" data set). In a first step we constructed a statistical parsimony haplotype network utilizing the program TCS 1.21 [55] for understanding the degree of populating admixing (see Goal 2). The analysis was set to run with a connection limit of 95%.

In order to assess the degree of populating admixing as well as possible demographic effects of watersheds in *O. h. robertsoni *(Goal 2) and to test different isolation-by-distance scenarios (Goal 3), we analysed the genetic structure by performing hierarchical AMOVAs. The significance of the Φ statistic was tested by generating null-distributions based on 10,000 permutations of the original data set using Arlequin 3.5.1.2 [56]. Hierarchical grouping variables were (i) between groups of populations, (ii) between populations, (iii) within populations.

An alternative test for group structure was performed using spatial analyses of molecular variance implemented in SAMOVA 1.0 [57] with significance being tested by 1,026 permutations. The optimal number of groups (*K*) was revealed by increasing *K *until the variance among geographically adjacent groups relative to the total variance (F_*CT*_) reached a maximum value [57].

Then, for inferring possible past demographic and spatial expansion events (Goal 2), we studied the demographic and spatial histories of *O. h. robertsoni *populations within those clades that did not exceed the 95% connection limit of the network analysis (see Result section). Utilizing mismatch analyses [58-60], we attempted to link the amount and distribution of sequence differences to relative time since divergence.

As a demographic expansion model requires panmixia (whereas a spatial expansion model does not), we first tested the null hypothesis of a random distribution of different haplotypes among populations (i.e., panmixia). Using the exact test as suggested by Raymond & Rousset [61] and implemented in Arlequin 3.5.1.2, significance was evaluated by running the Markov chain for 1,000,000 steps.

Finally, we attempted to detect possible correlations between genetic and geographic distances (see Goal 3), conducting simple Mantel's matrix correlation tests [62] with the geographical distance variables suggested in Table 4 and K2P genetic distances calculated in Arlequin 3.5.1. For all runs, the null hypothesis of no correlation between geographic and genetic distances was tested in TFPGA [63] with 10,000 permutations.

### Phylogeographical analyses (AFLP)

For AFLP-based phylogeographical analyses of *O. h. robertsoni *from the Deyang-Mianyang area, we used profiles from a total of 135 specimens.

First, we performed a NeighborNet analysis with the program SplitsTree 4.10 [64] in order to visualize relationships among AFLP genotypes for a better understanding of the degree of populating admixing (Goal 2). The NeighborNet algorithm, which is based on maximum likelihood genetic distances, was chosen here because empirical data show that it works well in resolving large or divergent data sets [64].

For studying the correlation of population structure and physical barriers (see Goal 2), we performed AMOVA and SAMOVA analyses similar to the ones conducted for our COI data set.

Finally, we attempted to test patterns of dispersal under different isolation-by-distance scenarios (Goal 3) by using Mantel tests based on Nei's [65] unbiased genetic distances calculated in Genalex 6.4 [66].

In addition, we applied a Mantel test to infer the correlation between genetic distances generated based on the COI gene and AFLP data.

## Competing interests

This work was supported in part by the NIH/NSF Ecology of Infectious Disease Program (grant no. 0622743), the National Institute for Allergy and Infectious Disease (grant no. K01AI091864), and by the Emory Global Health Institute Faculty Distinction Fund. The funders had no role in study design, data collection and analysis, decision to publish, or preparation of the manuscript. The views expressed in this article are solely those of the authors, and may not reflect the policies or views of the funding agencies.

## Authors' contributions

AKH: performed all genetic work and most phylogeographical analyses, helped with establishing the goals of the paper and with the writing, prepared the figures, and contributed to the discussion; JR: conceptualized and proposed the overall research, developed the overall study design, headed the specimen sampling, led the watershed analyses and contributed to the discussion; NX: contributed to the study design, assisted with the specimen sampling and preservation, and contributed to the discussion; GMD: helped in establishing the overall framework of the research, participated in establishing the sampling design and in the field work, and contributed to the discussion; LD: assisted with specimen sampling, sampling design, GPS mapping, and specimen preservation, and contributed to the discussion; MJB: performed the watershed analyses, calculated the geographical distance variables, and contributed to the writing; TW: drafted the paper, established the goals and hypotheses and conducted the phylogenetic as well as part of the phylogeographical analyses. All authors read and approved the final version of the manuscript.
